# Effects of nine weeks L-Carnitine supplementation on exercise performance, anaerobic power, and exercise-induced oxidative stress in resistance-trained males

**DOI:** 10.20463/jenb.2018.0026

**Published:** 2018-12-31

**Authors:** Majid S. Koozehchian, Amin Daneshfar, Ebrahim Fallah, Hamid Agha-Alinejad, Mohammad Samadi, Mojtaba Kaviani, Maryam Kaveh B, Y. Peter Jung, Mozhgan Hassanzadeh Sablouei, Najmeh Moradi, Conrad P. Earnest, T. Jeff Chandler, Richard B. Kreider

**Affiliations:** 1 Department of Kinesiology, Jacksonville State University, Alabama USA; 2 School of Health Science, University of Canterbury, Christchurch New Zealand; 3 Kinesiology, Tarbiat Modares University, Tehran Iran; 4 Exercise Physiology Research Center, Baqiyatallah University of Medical Science, Tehran Iran; 5 School of Nutrition and Dietetics, Acadia University, Wolfville Canada; 6 Department of Physical Education and Sport Sciences, Kharazmi University, Tehran Iran; 7 Department of Pharmacy Practice, Karnataka College of Pharmacy, Bangalore India; 8 Exercise & Sport Nutrition Lab, Texas A&M University, Texas USA; 9 Department of Kinesiology, Azad University, Tehran Iran; 10 Kinesiology, Azad University, Isfahan Iran

**Keywords:** strength, lactate, antioxidant

## Abstract

**[Purpose]:**

Studies of L-carnitine in healthy athletic populations have yielded equivocal results. Further scientific-based knowledge is needed to clarify the ability of L-carnitine to improve exercise capacity and expedite the recovery process by reducing oxidative stress. This study aimed to examine the 9-week effects of L-carnitine supplementation on exercise performance, anaerobic capacity, and exercise-induced oxidative stress markers in resistance-trained males.

**[Methods]:**

In a double-blind, randomized, and placebo-controlled treatment, 23 men (age, 25±2y; weight, 81.2±8.31 kg; body fat, 17.1±5.9%) ingested either a placebo (2 g/d, n=11) or L-carnitine (2 g/d, n=12) for 9 weeks in conjunction with resistance training. Primary outcome measurements were analyzed at baseline and at weeks 3, 6, and 9. Participants underwent a similar resistance training (4 d/w, upper/lower body split) for a 9-week period. Two-way ANOVA with repeated measures was used for statistical analysis.

**[Results]:**

There were significant increases in bench press lifting volume at wk-6 (146 kg, 95% CI 21.1, 272) and wk-9 (245 kg, 95% CI 127, 362) with L-carnitine. A similar trend was observed for leg press. In the L-carnitine group, at wk-9, there were significant increases in mean power (63.4 W, 95% CI 32.0, 94.8) and peak power (239 W, 95% CI 86.6, 392), reduction in post-exercise blood lactate levels (-1.60 mmol/L, 95% CI -2.44, -0.75) and beneficial changes in total antioxidant capacity (0.18 mmol/L, 95% CI 0.07, 0.28).

**[Conclusion]:**

L-carnitine supplementation enhances exercise performance while attenuating blood lactate and oxidative stress responses to resistance training.

## INTRODUCTION

L-carnitine (LCR) is an endogenous compound synthesized in mammals from the essential amino acids lysine and methionine^1^. LCR is primarily stored in skeletal muscles and the heart at approximately 95%, while significantly lower concentrations are stored in the plasma^1^. From a physiological standpoint, LCR serves as a substrate for the enzyme carnitine palmitoyltransferase as well as the synthesis of acetylcarnitine, which is necessary for maintaining a feasible pool of free coenzyme A (CoA), allowing for continuation of pyruvate dehydrogenase complex (PDC) and tricarboxylic acid cycle flux^2^. Theoretically, higher PDC flux during strenuous exercise would be expected to reduce blood lactate (BL) accumulation, which could potentially preserve glycogen stores and subsequently delay premature fatigue^3,4^. In addition, it is believed that LCR may reduce lactate production by maintaining the catalytic activity of the PDC through a buffering mechanism, thereby decreasing the acetyl-CoA/CoA ratio^5^. Siliprandi et al.^6^ reported that LCR reduced lactate accumulation which was attributed to the constant acetyl CoA/CoA ratio and continuous flux of PDC.

As a potent anti-inflammatory compound, LCR has been shown to significantly reduce the levels of inflammatory markers such as interleukin-6 (IL-6) and tumor necrosis factor-α (TNF- α)^7^ when used for long durations as a supplement; on the other hand, plasma levels of cytokines such as interleukin-1β (IL-1β), TNF-α, and IL-6 increase during and following intense prolonged exercise^8^. In particular, resistance training disrupts the balance between free radical production and the body’s antioxidant defense system, resulting in a condition called exercise-induced immune dysfunction^9^. 

LCR can also act as an antioxidant during recovery from exercise, thereby mitigating oxidative stress, which may then decrease exercise-induced muscle damage. Guzel et al.^10^ showed that 3 g of acute LCR supplementation increased glutathione and nitrate-nitrite levels identified as antioxidant markers after exhaustive exercise in young soccer players. Synergistic LCR supplementations with dietary choline and carnitine for a 21-d period has been shown to lower lipid peroxidation and promote conservation of retinol and α-tocopherol in healthy women before and after mild exercise^11^. Furthermore, 2 g/d of L-carnitine L-tartrate (LCLT) supplementation for 3 wk attenuated exercise-induced plasma markers of purine catabolism and circulating cytosolic proteins^12^. Magnetic resonance image scans in the same study indicated that muscle disruption in LCLT group was only 41-45% of the placebo area. LCLT supplementation appeared to mediate quicker recovery from hypoxic exercise^13^. Broad et al.^14^ found that 2 g/d of LCLT supplementation for 2 wk suppressed the plasma ammonia response, an indicator of metabolic stress, to exercise in non-vegetarian active men. Additionally, it was found that LCLT supplementation reduced muscle oxygenation responses to resistance training and attenuated plasma malondialdehyde (MDA), a marker of membrane damage^15^. Despite the popularity of resistance training and increased exercise-induced muscle damage, little attention has been paid to the potential benefits of LCR when combined with resistance training and whether it might improve exercise performance by reducing muscle damage markers. Therefore, the purpose of this study was to investigate the effects of 9-wk LCR supplementation on exercise performance, anaerobic performance, and exercise-induced oxidative stress in resistance-trained males. We hypothesized that, in comparison with PLA, supplementation with LCR would provide greater gains in strength, enhance anaerobic capacity, and improve recovery following a resistance training program.

## METHODS

### Participants

**Figure 1.**
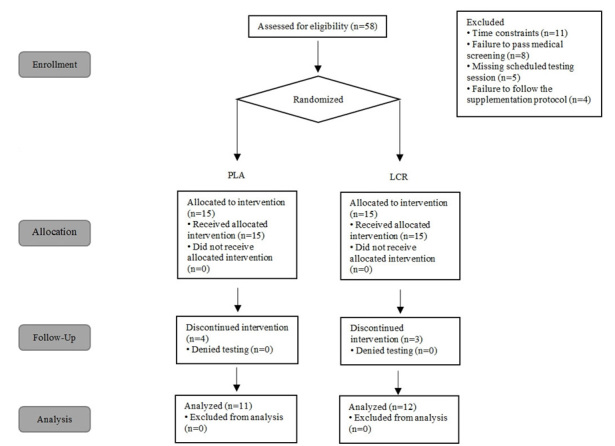
Consolidated standards of reporting trials diagram of study.

A diagram of the study enrollment is illustrated as a CONSORT (Fig. 1). Twenty-three men volunteered from Tarbiat Modares University and the local surrounding community to participate in this 9-wk study. Inclusion criteria were as follows: good health, aged 18-40 y, body fat percentage 10-25%, and at least one year of regular resistance training including bench press and leg press/squats. Exclusion criteria were current smoking habit or use of nutritional supplements, and any problems that might affect their ability to perform resistance training. Physical activity levels were determined using standardized questionnaires adapted from the Stanford Usual Activity Questionnaire, Baecke Physical Activity Questionnaire, Kent State University, and Eastern Michigan University at baseline and wk 3, 6, and 9. In the familiarization session, testing procedures and potential risks and benefits associated with the study were verbally explained in detail. Participants then provided written informed consent prior to participation in accordance with the guidelines established by the Institutional Review Board at Tarbiat Modares University (approval #: 7RT-0258).

### Experimental design

A placebo-controlled, double-blind design was used to conduct this study. All the testing was conducted in the exercise physiology laboratory at Tarbiat Modares University. Participants were matched into either the PLA or LCR group based on body mass, age, and resistance training experience. During the familiarization session and following informed consent, a research nutritionist and a professional strength and conditioning specialist met with each participant and explained in detail the strength training regimen as well as the nutritional and supplement requirements for the study period. 

### Testing sessions

**Figure 2.**
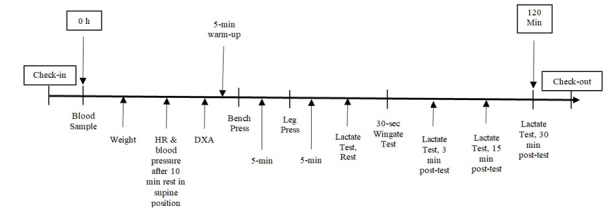
Presents the testing sequence timeline at baseline and weeks 3, 6, and 9.

The timeline of the testing protocol is presented in Fig. 2. The study included testing at baseline and at wk 3, 6, and 9, at which time blood samples were obtained, and body composition, exercise performance tests, and a series of BL tests were performed. Participants were instructed to refrain from strenuous exercise for 48 h and to have fasted for at least 12 h prior to each testing session.

### Stength assessment

In the familiarization session, upper and lower body muscular strength was assessed using an isotonic bench press and leg press (Pullum Power Sports, Luton, United Kingdom) to determine the 1-repetition maximum (1RM). The 1RM was determined following a standard warm-up including 10 repetitions using 50% of participants’ estimated 1RM, 5 repetitions using 70% of their estimated 1RM, and 1 repetition using 90% of their estimated 1RM. Weight was added until the 1RMs were determined. Verbal encouragement was provided during the test to ensure maximal effort. In the testing sessions, participants initially performed a general warm-up of ~5 min of light activity involving all muscles to be tested. Next, using the 1RM that was determined in the familiarization session, participants performed 3 sets of bench and leg press tests. For the first and second sets, participants performed 10 repetitions at 70% of 1RM on the bench press and leg press interspersed by 2 min of rest between sets and 5 min of recovery between each exercise. During the third set, participants were asked to complete as many repetitions as possible. Total lifting volume was calculated by multiplying the lifted weight times the number of completed repetitions. Test-retest reliability of performing upper and lower body strength assessments in our laboratory on resistance-trained participants showed low day-to-day mean coefficients of variation (CVs) and high reliability for the bench press (5.2%, intraclass, *r*=0.98) and leg press (7.4%, intraclass, *r*=0.97).

### Anaerobic capacity assessment

Participants underwent a Wingate test on a computerized Lode Sport Cycle Ergometer (Lode BV, Groningen, The Netherlands) equipped with toe clips at a standardized torque factor of 0.7. The torque factor was set based on the manufacturer’s guidelines relative to the population being tested. The seat position, seat height, handlebar position, and handlebar height were determined during familiarization sessions and repeated for all testing sessions. Participants were instructed to begin pedaling 10 s prior to application of the workload and continue at an all-out maximal capacity for the 30-s Wingate test. Test-retest reliability of performing Wingate test on participants in our laboratory yielded low day-to-day mean CVs and high reliability for absolute peak power (9.3%, intraclass, *r*=0.95) and mean power (7.6%, intraclass, *r*=0.94).

### Body composition

Body composition was determined by dual energy X-ray absorptiometry (DXA) (*Lunar Prodigy; General Electric, Waukesha, WI*). Quality control calibration and scanning procedures were conducted as previously described^16^. All participants were scanned in the morning in a fasted state. Mean test-retest reliability studies performed on male athletes in our lab with the DXA machine yielded low mean CVs for total bone mineral content and total fat-free/soft tissue mass of 0.31–0.45% with a mean intraclass correlation of 0.985.

### Blood lactate

BL levels were analyzed from finger prick capillary blood samples (*Analox GM7 Lactate Analyzer; Analox, Hammersmith, UK*). The analyzer device was calibrated using a standard control solution before each testing session. BL was measured 5 min prior to and immediately after the Wingate test and at 3, 15, and 30 min. The test-to-test reliability of conducting BL tests in our laboratory on resistance-trained males indicated low day-to-day mean CV and high reliability (5.2%, intraclass, *r*=0.89).

### Resting heart rate & blood pressure

Resting heart rate (RHR) was measured after 10 min of rest in the supine position using standard procedures^17^. Then, systolic blood pressure (SBP) and diastolic blood pressure (DBP) were determined by auscultation of the brachial artery and a mercurial sphygmomanometer, based on standard clinical procedures^17^.

### Blood collection

Venous blood samples of approximately 10 mL were drawn after fasting for 12 h at the beginning of each testing session. Samples were collected from the antecubital vein in two 7.5-mL collection tubes utilizing a standard vacutainer apparatus. Blood samples were kept at room temperature for 15 min and then centrifuged at 3500 rpm for 10 min. The serum supernatant was removed and stored at -80℃ in polypropylene microcentrifuge tubes for later analysis. 

### Serum clinical chemistry analyses

Laboratory measures were conducted at baseline, and weeks 3, 6, and 9. The tests included total and free carnitine, total antioxidant capacity (TAC), MDA, glutathione peroxidase (GPx), superoxide dismutase (SOD), catalase (CAT), IL-6, and TNF-α. All blood samples were analyzed in a biochemistry laboratory located at Tarbiat Modares University in Tehran, Iran. Day-to-day variability of the oxidative stress markers in our lab yielded a CV range of 0.06-0.23 and an intraclass correlation coefficient range of 0.67-0.90. 

### Supplementation protocol and dietary monitoring

Using a randomization code in a double-blind, placebo-controlled manner, participants in both the LCR and PLA groups were orally administered either 2 g/d-1 of LCR (Sina Nutrition, Inc., Tehran, Iran) or PLA (maltodextrin) for a 9-wk period. Both the LCR and PLA supplements were in the form of identical-looking ingestible capsules. Participants were instructed to consume 1 capsule with breakfast and 1 capsule with lunch (1 g per serving). The use of this dose has been shown to be safe and efficacious in previous studies^13,18,19^. Supplementation began ~30 min after the baseline testing session and continued throughout the 9-wk period. Compliance to the supplementation protocol was monitored by the research dietician who contacted participants on a weekly basis by phone. Participants were also asked to return all empty containers to the testing sessions at wk 3, 6, and 9, which allowed study personnel to assess compliance with the protocol.

Participants were instructed to maintain their current dietary intake throughout the study. In addition, they were given instructions during the familiarization session on how to record portion sizes and quantities. Participants completed a 3-day food recall (i.e., 2 weekdays and 1 weekend day) 1 week before all testing sessions. Dietary records were analyzed for total kilocalories, carbohydrate, protein, and fat using the NutraBase IV Clinical Edition (*CyberSoft, Inc., Phoenix, AZ*).

### Resistance training protocol

Participants in both the PLA and LCR groups completed a 4-day/week resistance training program previously described in detail^20^. The weekly training volume was the same between the LCR and PLA groups. Briefly, the protocol involved training the upper and lower body twice per week using a 4-day split (i.e., upper body1, lower body1, upper body2, lower body2). The training program was composed of 15 exercises, including bench press, lat pulldown, shoulder press, seated row, dips, pullover, biceps curl, triceps press down, leg press, leg extension, leg curl, back extension, half squat, standing calf raise, and stiff leg deadlift. For each exercise, participants performed 3-6 sets of 8-15 repetitions with as much weight as they could while maintaining a proper form. 

Further, participants maintained their training intensity between 70-85% of 1RM throughout the study. Rest periods between exercises were 1-2 min. Two certified strength and conditioning specialists supervised all lifts and showed participants how to record training data (i.e., lifts performed, reps, amount of weight lifted, etc.). Training was performed at 3 different training facilities, recorded in training logs, and signed off by selected fitness instructors to verify compliance. All 3 sports clubs used identical training equipment. Furthermore, at each testing session, participants were required to complete a physical activity questionnaire, describing their physical activity during the previous month.

### Biochemical analyses

LCR fraction in all samples was analyzed by SRL Inc. (Tokyo, Japan). Total and free LCR levels were measured using an enzyme cycling method with an autoanalyzer (JCA-BM8000 series; JEOL, Tokyo, Japan)^21^. TAC was measured as previously described by Erel et al.^22^ and reported in mmol/L. MDA was measured using the method described by Vassalle et al.^23^ and expressed in μmol/L. GPx activity was measured using the method described by Bulucu et al.^24^ and expressed in U/mL. SOD activity was measured as the inhibition of the rate of reduction of cytochrome c by the superoxide radical, observed at 550 nm as previously described by Berzosa et al.^25^; it was reported in μmol/mL. The CAT activity was measured in hemolysates as described by Aebi et al.^26^. and reported in nmol/mL. Serum TNF-α and IL-6 levels were measured by enzyme-linked immunosorbent assay (ELISA) technique as previously described by Arican et al.^27^. TNF-α and IL-6 activities were reported in pg/mL.

### Adverse events

Study-related side effects were assessed using a questionnaire completed at each study visit. Participants reported how well they tolerated the supplement, how well participants followed the supplementation protocol, and whether participants encountered any medical issues and/or adverse symptoms throughout the study. The questionnaire consisted of the following 13 supplement-related symptoms: abdominal or stomach cramps, diarrhea, headache, nausea or vomiting, abdominal discomfort, body odor, depression, dizziness, impaired vision, loss of appetite or weight, swelling in hands or lower legs and feet, tingling sensation, and weakness. The options for each symptom were not at all, somewhat, moderately, very much, or extremely. Participants were asked to rank the frequency and severity of their symptoms during the supplementation period. 

### Statistical analysis

Data were analyzed using two-way ANOVA with repeated measures, evaluating for between-group differences as well as changes from baseline in body composition, HR and blood pressure, exercise performance, and blood markers. Data were considered statistically significant when the probability of error was 0.05. Data are presented as mean ± SD or mean change ± 95% CI as appropriate.

## RESULTS

### Participant demographics

The demographic characteristics of the groups are presented in Table 1. Thirty male participants were initially recruited for the study. Of these, 7 participants withdrew from the study due to personal reasons, and 3 were excluded due to low compliance (<80%) to the supplement. Therefore, a total of 23 participants completed the study. Characteristics of the study participants are presented in Table 1.

**Table 1.** Baseline characteristics of the study participants.

	Group	Mean
Age (y)	PLA	24.5 ± 1.5
	LCR	25.5 ± 1.5
Height (cm)	PLA	170.4 ± 5.8
	LCR	171.3 ± 3.1
Weight (kg)	PLA	77.9 ± 6.8
	LCR	84.1 ± 8.7
Body mass index	PLA	26.6 ± 3.4
	LCR	28.7 ± 3.5
Body fat (%)	PLA	16.1 ± 5.7
	LCR	18.0 ± 6.0
Resting HR (b/min)	PLA	57.0 ± 5.5
	LCR	60.5 ± 7.8
Resting SBP (mmHg)	PLA	116.1 ± 5.9
	LCR	114.5 ± 5.3
Resting DBP (mmHg)	PLA	77.2 ± 3.9
	LCR	74.0 ± 5.3

Values are means ± standard deviations. Data for the PLA (n= 11) and LCR (n=12) groups were analyzed by one-way ANOVA.

### Dietary analysis, supplement & training compliance, and reported side effects

Food logs were used to measure the average daily caloric and macronutrient intake (Table 2). No significant difference in total calorie, protein, fat, and carbohydrate intake was observed among groups (*p*>0.05). Furthermore, subjective assessment of the physical activity evaluations indicated that none of the participants had any prominent changes in their level of physical activity throughout the 9 wk.

**Table 2.** Dietary and anthropometric characteristics of study participants

	Group	Time (wk)					p-level
		Week 0	Week 3	Week 6	Week 9		
		Mean ± SD	Mean ± SD	Mean ± SD	Mean ± SD		
**Diet Characteristics**							
Energy Intake (kcals/day)	PLA	2,116 ± 718	2,147 ± 723	2,250 ± 546	2,000 ± 311	G x T	0.48
	LCR	2,449 ± 529	2,414 ± 490	2,457 ± 549	2,444 ± 439		
Protein (g)	PLA	145.9 ± 38.5	151.9 ± 44.2	153.6 ± 44.0	156.4 ± 59.3	G x T	0.57
	LCR	147.7 ± 37.4	156.3 ± 39.1	157.1 ± 38.2	162.9 ± 46.1		
Fat (g)	PLA	74.4 ± 36.7	72.2 ± 35.3	73.5 ± 33.1	74.2 ± 26.0	G x T	0.73
	LCR	93.4 ± 32.1	98.8 ± 28.3	96.2 ± 22.8	95.0 ± 25.7		
Carbohydrate (g)	PLA	198.9 ± 68.1	202.0 ± 50.3	218.2 ± 70.2	185.1 ± 32.1	G x T	0.46
	LCR	258.5 ± 106.0	231.2 ± 81.2	240.7 ± 91.0	218.9 ± 61.0		
**Anthropometry**							
Body Weight (kg)	PLA	77.9 ± 7.09	78.1 ± 7.12	77.6 ± 7.26	78.1 ± 7.36	G x T	0.10
	LCR	84.3 ± 8.98	84.5 ± 8.78	84.3 ± 8.85	83.7 ± 8.92		
Fat Mass (kg)	PLA	12.1 ± 5.05	12.2 ± 5.29	12.0 ± 5.34	12.2 ± 5.14	G x T	0.15
	LCR	14.8 ± 5.26	15.1 ± 4.94	14.5 ± 4.73	14.2 ± 4.74		
Fat-Free Mass (kg)	PLA	54.1 ± 2.70	54.2 ± 2.70	54.0 ± 2.67	54.2 ± 2.63	G x T	0.06
	LCR	56.2 ± 2.78	56.3 ± 2.67	56.1 ± 2.56	56.8 ± 2.70		

Values are means ± standard deviations. Dietary intake data were analyzed by two-way ANOVA with repeated measures. Greenhouse-Geisser group (G), time (T), and group x time (G x T) interaction p-levels are reported with univariate treatment p-levels. The analysis revealed the overall Wilks’ Lambda group (p=0.17), time (p=0.07), and group x time (p=0.44) effects.

### Body composition

Body composition data is shown in Table 2. No significant differences were observed between groups for the components of body composition (Wilks’ Lambda group *p*=0.31, time *p*=0.02, and group x time *p*=0.06). Univariate analysis indicated that LCR supplementation did not influence body weight, fat mass, or fat-free mass compared to the PLA group (*p*>0.05).

### Perfomance assessment: Muscular strength

**Figure 3.**
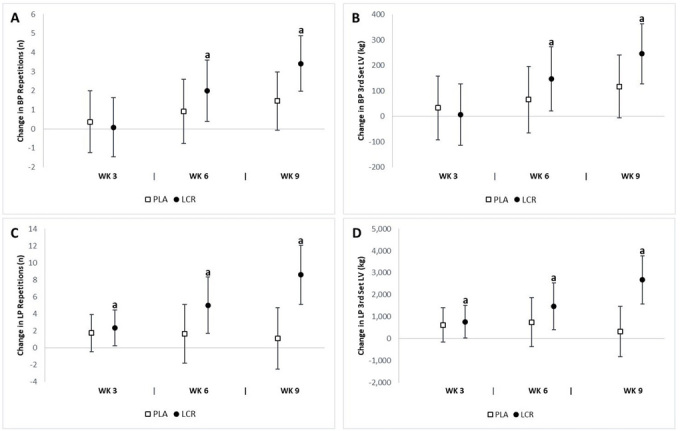
Change in strength performance for the placebo (PLA) and L-carnitine (LCR) treatments at baseline and weeks 3, 6, and 9. Panels A & B represent the change in bench press (BP) repetitions and third set lifting volume (LV), respectively. Panels C & D represent change in leg press (LP) repetitions and third set lifting volume, respectively. (a) denotes a statistically significant change from baseline (p<0.05). Values are the mean change ± 95% confidence interval.

Bench press. Results for all exercise performance variables are presented in Table 3. The analysis did not reveal a significant interaction effect between groups in the bench press performance (*p*>0.05). However, the analysis using baseline values as a covariate and evaluation of the mean change and 95% CIs of the 1RM upper body strength data demonstrated a significant increase in bench press performance (Fig. 3 A & B). The number of reps significantly increased at week 6 only in the LCR group (2.00 n, 95% CI, 0.39, 3.60) but not in the PLA group (0.90 n, 95% CI -0.77, 2.59). For week 9, the bench press reps assessment was as follows: LCR (3.41 n, 95% CI 1.96, 4.87), PLA (1.45 n, 95% CI -0.06, 2.97). A significant change in the bench press third set lifting volume at week 6 was observed in the LCR group (146 kg, 95% CI 21.1, 272) but not in the PLA group (65.2 kg, 95% CI -65.7, 196). For week 9, the bench press third set lifting volume was as follows: LCR (245 kg, 95% CI 127, 362), PLA (117 kg, 95% CI -5.64, 239). The percent changes from baseline in BP reps and third set lifting volume were both 27.5% for the LCR group.

**Table 3.** Exercise performance characteristics of study participants

	Group	Time (wk)					p-level
		Week 0	Week 3	Week 6	Week 9		
		Mean ± SD	Mean ± SD	Mean ± SD	Mean ± SD		
Bench Press Repetitions (n)	PLA	12.0 ± 3.3	12.3 ± 2.9	12.9 ± 3.4	13.4 ± 3.2	G x T	0.08
	LCR	14.1 ± 4.0	14.2 ± 4.2	16.1 ± 3.8	17.5 ± 4.1		
Bench Press third Set Lifting Volume (kg)	PLA	1,042 ± 374	1,075 ± 335	1,107 ± 321	1,159 ± 333	G x T	0.17
	LCR	1,005 ± 315	1,012 ± 311	1,152 ± 296	1,250 ± 306		
Leg Press Repetitions (n)	PLA	22.7 ± 8.32	24.4 ± 9.16	24.3 ± 7.7	23.8 ± 9.0	G x T	0.01
	LCR	26.0 ± 6.92	28.4 ± 8.79	31.0 ± 7.4	34.6 ± 7.59		
Leg Press third Set Lifting Volume (kg)	PLA	9,032 ± 3,556	9,665 ± 3,784	9,788 ± 4,036	9,364 ± 3,733	G x T	0.01
	LCR	8,662 ± 3,553	9,440 ± 4,062	10,145 ± 3,210	10,836 ± 3,835		
Wingate Mean Power (Watts)	PLA	545 ± 85	524 ± 76	553 ± 75	540 ± 92	G x T	0.08
	LCR	545 ± 85	553 ± 133	586 ± 120	624 ± 120		
Wingate Peak Power (Watts)	PLA	1,639 ± 303	1,580 ± 345	1,633 ± 388	1,595 ± 441	G x T	0.03
	LCR	1,712 ± 363	1,751 ± 329	1,755 ± 302	1,952 ± 424		
Wingate Absolute Peak Power (Watts)	PLA	21.2 ± 4.97	20.4 ± 5.34	21.1 ± 5.28	20.6 ± 6.51	G x T	0.04
	LCR	20.5 ± 4.70	20.9 ± 4.48	20.8 ± 4.02	23.2 ± 5.20		
Wingate Relative Peak Power (Watts/kg)	PLA	7.00 ± 0.82	6.73 ± 1.03	7.13 ± 0.74	6.91 ± 0.92	G x T	0.10
	LCR	6.78 ± 1.92	7.67 ± 2.02	7.95 ± 1.87	8.49 ± 1.82		

Values are means ± standard deviations. Bench press, leg press, and cycling performance data were analyzed by two-way ANOVA with repeated measures. Greenhouse-Geisser group (G), time (T), and group x time (G x T) interaction p-levels are reported with univariate treatment p-levels. The analysis revealed the overall Wilks’ Lambda group (p=0.03), time (p<0.0001), and group x time (p=0.02) effects.

Leg press. The number of leg press reps increased in the LCR group compared to the PLA group (*p*=0.01). In addition, the leg press third set lifting volume increased in the LCR compared to the PLA group (*p*=0.01). The analysis of mean changes with 95% CIs demonstrated significant differences in lower body performance between groups (Fig. 3 C & D). The change in leg press reps from baseline to week 6 was as follows: LCR (5.00 n, 95% CI, 1.67, 8.32), PLA (1.63 n, 95% CI, -1.83, 5.10). The change in leg press reps at week 9 was as follows: LCR (8.58 n, 95% CI 5.09, 12.06), PLA (1.09 n, 95% CI -2.54, 4.73). 

Comparisons at week 3 demonstrated a significant increase in leg press third set lifting volume in the LCR group (777 kg, 95% CI, 32.3, 1523) but not in the PLA group (633 kg, 95% CI -145, 1,411). There was a significant mean change from baseline to week 6 in the LCR group (1,483 kg, 95% CI 416, 2,549) but not in the PLA group (756 kg, 95% CI -357, 1,870). A significant improvement was observed at week 9 only in the LCR group (2,683 kg, 95% CI 1,591, 3,774) but not in the PLA group (331 kg, 95% CI -808, 1,471). The percent changes from baseline in LP reps and third set lifting volume in the LCR group were 38.1% and 30.2%, respectively.

### Anaerobic power

**Figure 4.**
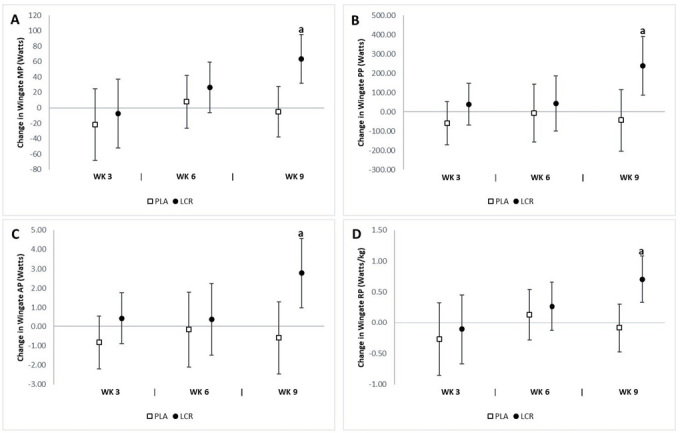
Change in cycling test performance for the placebo (PLA) and L-carnitine (LCR) treatments at baseline and weeks 3, 6, and 9. Panels A, B, C, and D represent the change from baseline in mean power (MP), peak power (PP), absolute peak power (AP), and relative peak power (RP), respectively. (a) denotes statistically significant change from baseline (p<0.05). Values are mean change ±95% confidence interval.

The analysis revealed significant interaction effects for peak power (*p*=0.03) and absolute peak power (*p*=0.04) between groups, but no significant interaction effect in mean power or relative peak power (*p*>0.05) between groups. The analysis of mean changes with 95% CIs indicated significant differences in anaerobic performance between groups (Fig. 4). There was a significant improvement in mean power at week 9 in the LCR group (63.4 Watts, 95% CI 32.0, 94.8) but not in the PLA group (-5.24 Watts, 95% CI -38.0, 27.5). A significant change in peak power at week 9 was observed in the LCR group (239 Watts, 95% CI 86.6, 392) but not in the PLA group (-43.5 Watts, 95% CI -203, 116); the significant change in absolute peak power at week 9 was also observed in the LCR group (2.78 Watt/kg, 95% CI 0.99, 4.57) but not in the PLA group (-0.59 Watt/kg, 95% CI -2.46, 1.27). A significant change in relative power at week 9 was observed in the LCR group (0.70 Watt/kg, 95% CI 0.33, 1.08) but not in the PLA group (-0.08 Watt/kg, 95% CI -0.47, 0.30). In the LCR group, the percent changes from baseline in mean power, peak power, absolute peak power, and relative peak power were 12.8%, 14.8%, 12.5%, and 14.1%, respectively.

### Total and free l-carnitine assessment

**Figure 5.**
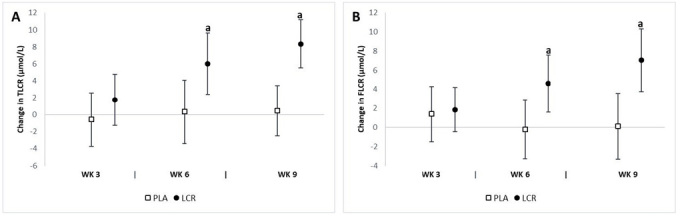
Change in plasma LCR levels for the placebo (PLA) and L-carnitine (LCR) treatments at baseline and weeks 3, 6, and 9. Panels A and B represent the total LCR (TLCR) and free LCR (FLCR) levels, respectively. (a) denotes a statistically significant change from baseline (p<0.05). Values are mean change ± 95% confidence interval.

We observed significant differences between groups in both the total (*p*=0.005) and free (*p*=0.003) LCR levels. The analysis of mean changes with 95% CI’s indicated significant changes in total and free LCR levels between groups (Fig. 5). Significant mean changes from baseline in total plasma LCR levels at week 6 were seen in the LCR group (6.02 μmol/L, 95% CI 2.42, 9.63) but not in the PLA group (0.33 μmol/L, 95% CI -3.43, 4.09). A significant increase in total plasma LCR levels at week 9 was observed in the LCR group (8.35 μmol/L, 95% CI 5.53, 11.1) and not in the PLA group (0.45 μmol/L, 95% CI -2.48, 3.40). Significant mean changes from baseline in free plasma LCR levels at week 6 were seen in the LCR group (4.59 μmol/L, 95% CI 1.62, 7.56) but not in the PLA group (-0.19 μmol/L, 95% CI -3.29, 2.90). Furthermore, free plasma LCR levels at week 9 were higher in the LCR group (7.04 μmol/L, 95% CI 3.74, 10.3) than in the PLA group (0.13 μmol/L, 95% CI -3.31, 3.57). The percent changes from baseline in total and free plasma LCR levels for the LCR group were 15.7% and 14.9%, respectively.

### Blood lactate & oxidative stress assessment

**Figure 6.**
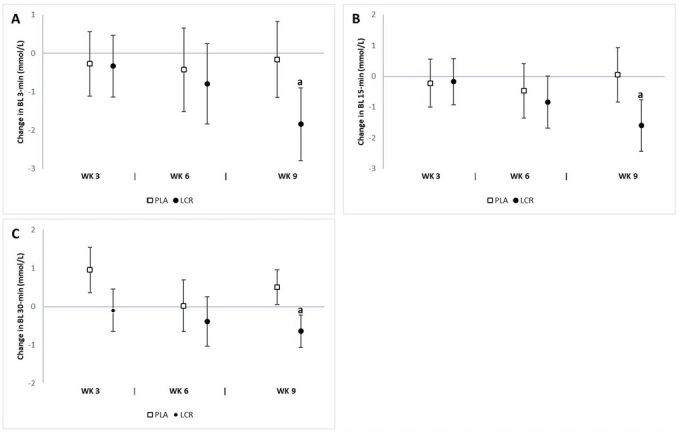
Change in post-exercise blood lactate (BL) levels for the placebo (PLA) and L-carnitine (LCR) treatments at baseline and weeks 3, 6, and 9. Panels A, B, and C represent post-exercise BL levels at minutes 3, 15, and 30, respectively. (a) denotes a statistically significant change from baseline (p<0.05). Values are mean change ± 95% confidence interval.

Table 4 presents the pre- and post-exercise BL assessments. The analysis revealed significant interaction effects for 3-min (*p*=0.04), 15-min (*p*=0.01), and 30-min (*p*=0.04) post-exercise BL levels. The analysis of mean changes with 95% CIs demonstrated significant changes in post-exercise BL levels between groups (Fig. 6). A significant decrease in 3-min post-exercise BL at week 9 was observed in the LCR group (-1.84 mmol/l, 95% CI -2.97, -0.90) and not in the PLA group (-0.17 mmol/L, 95% CI -1.15, 0.81). Significant mean changes from baseline in 15-min post-exercise BL at week 9 were seen in the LCR group (-1.60 mmol/L, 95% CI -2.44, -0.75) but not in the PLA group (0.04 mmol/l, 95% CI -0.83, 0.92). The mean change in 30-min post-exercise BL from baseline to week 9 was as follows: LCR (-0.64 mmol/L, 95% CI -1.07, -0.21), PLA (0.50 mmol/l, 95% CI 0.54, 0.95). The percent change from baseline at 3-min post-exercise BL level was as follows: LCR (-17.2%) and PLA (-1.46%); at min-15: LCR (-14.8%) and PLA (0.89%); and at min-30: LCR (-13.6%) and PLA (9.54%). 

**Table 4.** Post-exercise blood lactate and oxidative stress characteristics of the study participants.

	Group	Time (wk)					p-level
		Week 0	Week 3	Week 6	Week 9		
		Mean ± SD	Mean ± SD	Mean ± SD	Mean ± SD		
**Blood Lactate**							
3-min post-Wingate test (mmol/L^-1^)	PLA	10.7 ± 1.19	10.4 ± 1.47	10.2 ± 1.93	10.5 ± 2.04	G x T	0.04
	LCR	10.1 ± 0.99	9.80 ± 0.91	9.34 ± 1.46	8.29 ± 0.56		
15-min post-Wingate test (mmol/L^-1^)	PLA	10.7 ± 1.06	10.5 ± 1.64	10.3 ± 1.25	10.8 ± 1.24	G x T	0.01
	LCR	9.87 ± 1.49	9.70 ± 0.89	9.03 ± 0.92	8.27 ± 0.71		
30-min post-Wingate test (mmol/L^-1^)	PLA	5.73 ± 1.14	6.69 ± 0.98	5.75 ± 1.14	6.24 ± 1.25	G x T	0.04
	LCR	4.60 ± 0.97	4.51 ± 0.96	4.22 ± 1.27	3.96 ± 0.99		
**Oxidative Stress**							
TAC (mmol/L)	PLA	1.45 ± 0.22	1.41 ± 0.16	1.46 ± 0.19	1.43 ± 0.16	G x T	0.02
	LCR	1.49 ± 0.13	1.60 ± 0.10	1.66 ± 0.15	1.77 ± 0.14		
MDA (μmol/L)	PLA	0.64 ± 0.13	0.63 ± 0.18	0.62 ± 0.10	0.66 ± 0.14	G x T	0.02
	LCR	0.56 ± 0.15	0.47 ± 0.09	0.48 ± 0.16	0.31 ± 0.18		
GPx (U/mL)	PLA	11.9 ± 2.15	12.1 ± 2.21	11.9 ± 1.81	11.4 ± 2.05	G x T	0.03
	LCR	11.7 ± 2.23	12.1 ± 1.92	12.2 ± 1.50	13.5 ± 1.73		

Values are means ± standard deviations. Oxidative stress data were analyzed by two-way ANOVA with repeated measures. Greenhouse-Geisser group (G), time (T), and group x time (G x T) interaction p-levels are reported with univariate treatment p-levels. The analysis revealed the overall Wilks’ Lambda group (p=0.056), time (p=0.003), and group x time (p=0.004) effects.

**Figure 7.**
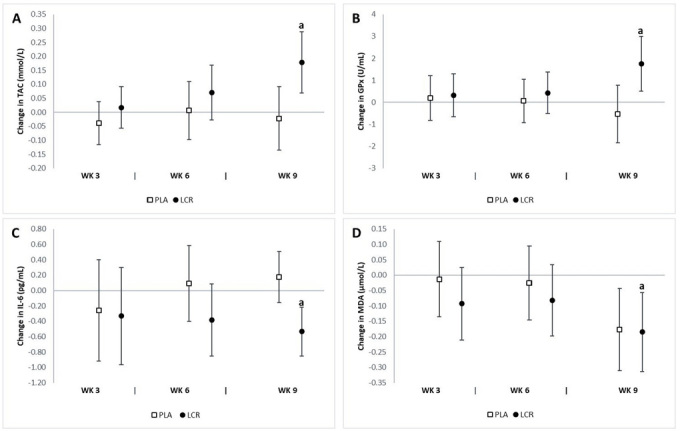
Change in oxidative stress status for the placebo (PLA) and L-carnitine (LCR) treatments at baseline and weeks 3, 6, and 9. Panels A, B, C, and D represent total antioxidant capacity (TAC), glutathione peroxidase (GPx), interleukin-6 (IL-6), and malondialdehyde (MDA), respectively. (a) denotes a statistically significant change from baseline (p<0.05). Values are mean change ±95% confidence interval.

The analysis revealed a significant interaction effect between groups in serum TAC (*p*=0.02), MDA (*p*=0.02), and GPx (*p*=0.03). We did not observe any significant difference in serum SOD, CAT, IL-6, or TNF-α levels between groups. The analysis of mean changes with 95% CIs demonstrated significant differences in oxidative stress biomarkers between groups (Fig. 7). There was a significant increase in serum TAC at week 9 in the LCR group (0.18 mmol/L, 95% CI 0.07, 0.28) but not in the PLA group (-0.02 mmol/L, 95% CI -0.13, 0.09). A significant increase was observed in serum GPx at week 9 in the LCR group (1.75 U/mL, 95% CI 0.49, 3.00) but not in the PLA group (-0.54 U/mL, 95% CI -1.85, 0.77). There was a significant decrease in serum IL-6 at week 9 in the LCR group (-0.53 pg/mL, 95% CI -0.85, -0.21) but not in the PLA group (0.17 pg/mL, 95% CI -0.15, 0.50). The percent changes from baseline in serum TAC and GPx for the LCR group were 11.5% and 17.4%, respectively.

## DISCUSSION

The main finding of our study was a significant increase in the BP and LP lifting volume at week 6 and week 9 in the LCR group. In addition, we observed a significant increase in mean power and peak power during the Wingate test. We further examined the effects of LCR on the metabolic response to exercise and found a significant attenuation in BL and markers of post-exercise inflammation. Interestingly, the observed changes in strength findings became manifest at week 6, while the Wingate and metabolic responses became significant at week 9. 

There are limited data regarding the underlying mechanisms of LCR supplementation in relation to enhanced muscle mass and strength^28^. Our results showed that LCR supplementation had no significant influence on muscle mass though it improved upper/lower body strength. The applied training program was previously reported to elicit myofibrillar protein synthesis and recruitment of fast-twitch motor units^20^; however, our results failed to report any significant difference in muscle mass between the experimental groups. This is in line with previous evidence indicating that despite a greater growth potential in type I fibers, hypertrophy response is limited compared to type II fibers^29^. Sawicka et al.^30^ showed that 8 weeks of LCR supplementation combined with creatine, L-leucine, and vitamin D resulted in an increase in muscle mass and strength due to elevated activation of the mTOR pathway. However, once LCR was tested alone using the same dosage, but for a longer period (i.e., 24 wk), no significant effect was found. In the present study, the training volume was significantly higher in the LCR group versus the PLA group. This may be attributed to the nature of the training program with moderate intensity, wherein the oxidation of long chain fatty acids acts as the predominant source of energy and LCR could increase the fat oxidation rate, thereby preserving muscle glycogen stores (25).

We reported that there was a significant reduction in BL accumulation post-30-sec Wingate test. In agreement with this, Jacobs et al.^31^ showed a reduced BL accumulation after only short (10-s) bouts of anaerobic tests where LCR was ingested in a single dosage. A longer duration of supplementation could be speculated to buffer hydrogen ions produced by lactic acid breakdown to a greater extent, resulting in less pronounced blood acidity^2,32^. In another attempt, Siliprandi et al.^6^ investigated the effects of 2 g of LCR before high-intensity exercise and found a decrease in plasma lactate, which may have been due to increased stimulation of pyruvate dehydrogenase activity. In contrast, Barnett et al.^5^ showed that LCR supplementation for 14 days had no significant effect on lactate accumulation following a high-intensity cycling performance, despite a significant increase in plasma free carnitine concentrations. The attenuation in BL concentrations after strenuous exercise combined with LCR supplementation appears to be primarily due to carnitine-mediated enhancement of PDC activation and flux. During exercise of this nature, when the use of the acetyl group via the Krebs cycle is exceeding its production by the PDC reaction, carnitine buffers against acetyl-CoA accumulation by making acetylcarnitine in an enzymatic reaction, thereby providing free Co-enzyme A to maintain the Krebs cycle flux^33^. 

L-carnitine is involved in the transportation of activated long-chain fatty acids from the cytosol into the mitochondrion and the buffering of acetyl-CoA^5^; therefore, LCR is essential for mitochondrial β-oxidation^34^. It has been hypothesized that the buffering action of LCR, which attenuates the acetyl-CoA/CoA ratio, may reduce lactic acid production by maintaining the catalytic activity of the PDC^5^. These conditions explain the impact of carnitine on lactic acid metabolism. 

The findings of our study also demonstrated that chronic LCR supplementation (2 g/d) increased TAC and GPx markers while it decreased MDA levels. Since no significant changes were observed in dietary intake during the study period, the changes in these markers may be attributed to the antioxidant capacity of LCR. Recent studies have indicated that LCR administration may prevent exercise-induced oxidative stress by decreasing lipid peroxidation, scavenging oxygen radicals, and upregulating the activities of antioxidant enzymes such as GPx, SOD, and CAT^10,35-37^. Lee et al.^19^ indicated that LCR might exert antioxidant properties for exercise-induced oxidative stress. After 3 wk of LCLT supplementation (2 g/d LCR), plasma MDA returned to resting values by 15 min post-exercise in the LCLT group, whereas MDA remained significantly elevated above pre-exercise levels throughout 24 h of recovery in the PLA group. Another study assessed the effect of 2 wk of LCR supplementation (2 g/d) on oxidative stress in active, healthy young men. Results indicated increased TAC and decreased serum MDA in the LCR group compared to the PLA group^36^. Inflammatory responses induce the production of reactive oxygen species (ROS), which regulate the expression of proinflammatory cytokines such as IL-1, IL-6, and TNF-α and subsequently activate the nuclear transcription factor-κB (NF-κB) pathway^38,39^. NF-κB, as a transcriptional regulator of DNA, plays a crucial role in the expression of more than 200 genes involved in immune and inflammatory responses^40,41^. Some studies identified both continuous and high-intensity intermittent exercise protocols as a strong stimulus of NF-κB activation^42-44^. Previous studies have shown that supplementation with antioxidants such as LCR, glutathione, and astaxanthin may reduce the formation of ROS, resulting in inhibition of the NF-κB activation cascade^45-48^.

### Conclusion and practical applications

A strength of our study was the duration of the intervention. Supplementing for this length of time helped to delineate the treatment effects; although strength performance improved by week 6, prolonged supplementation was necessary to observe the effects on anaerobic performance. Moreover, our findings were further strengthened by the fact that we recruited participants with 1 year of training experience, thus minimizing any neurological training effects and enhancing the generalizability of our study to individuals engaged in resistance training across various athletic disciplines. Hence, our results add to the known body of literature as LCR has been well studied in endurance athletes, but less is known regarding its effects on those involved in resistance training. A limitation of our study was the absence of muscle biopsy, which could have provided additional data regarding intramuscular LCR levels as well as molecular and cellular responses, including proteins involved in the mTOR pathway. Another limitation was the lack of measuring the stress factors related to the hypothalamus-pituitary-adrenal axis such as corticosterone, which may have helped explain the possible neurophysiological impact of LCR supplementation. From a practical point of view, our results suggested that 2 g/d of LCR supplementation improved muscle strength and anaerobic performance while decreasing post-exercise BL levels and attenuating exercise-induced oxidative stress markers in resistance-trained athletes. However, all of the abovementioned changes occurred independently of any change in body composition or hemodynamic parameters.
